# Factors associated with quality of life of outpatients with breast cancer and gynecologic cancers and their family caregivers: a controlled study

**DOI:** 10.1186/1471-2407-7-102

**Published:** 2007-06-19

**Authors:** Abdel W Awadalla, Jude U Ohaeri, Abdullah Gholoum, Ahmed OA Khalid, Hussein MA Hamad, Anila Jacob

**Affiliations:** 1 Department of Psychiatry, Faculty of Medicine, Kuwait University, Kuwait; 2 Department of Psychiatry, Psychological Medicine Hospital, Gamal Abdul Naser Road, P.O. Box 4081, Safat, 13041, Kuwait; 3 Radiation and Isotopes Centre, Khartoum (RICK), Khartoum, Sudan

## Abstract

**Background:**

Quality of life (QOL) issues are of interest in cancer because effective methods of treatment and detection have led to an increase in the number of long-term survivors. The objectives of the study were: to assess the subjective QOL of stable Sudanese women cancer outpatients and their family caregivers, using the WHO 26-item QOL Instrument; compare with matched general population groups, as well as diabetic and psychiatric patient groups; examine patient-caregiver concordance in ratings; and assess the variables associated with their QOL, with a view to identifying factors that can enhance quality of care.

**Methods:**

Responses of oncology outpatients with breast cancer (117), cervical cancer (46) and ovarian cancer (18) (aged 44.6, SD 11.5) were compared with those of their family caregivers and matched general population groups. Data were analyzed by univariate and multivariate statistics.

**Results:**

The cancer groups had similar QOL domain scores, which were significantly lower than those of their caregivers, but higher than the control group as well as those of psychiatric and diabetic patients studied previously. Patients who were married, with higher education, better employment, and with longer duration of illness had higher QOL. Patients on radiotherapy and their caregivers had higher QOL scores. Correlations between patient's ratings and caregiver impression of patient's QOL were high. Caregiver impression was a significant predictor of patient's and caregiver's QOL. Other predictors for the patient were: currently feeling sick and duration of illness; for the caregiver: feeling sick, relationship to patient, and age.

**Conclusion:**

Cancer patients in stable condition and with psychosocial support can hope to enjoy good QOL with treatment. The findings constitute an evidence base for the country's cancer care program, to boost national health education about prognosis in cancer. Families living with women cancer patients are vulnerable and need support if the patient is recently diagnosed, less educated, single, not formally employed; and the caregiver is female, parent, younger, less educated, unemployed and feels sick. Clinicians need to invest in the education and support of family caregivers. The patient-caregiver dyad should be regarded as a unit for treatment in cancer care.

## Background

Quality of life (QOL) issues are of interest in oncology because effective modern methods of treatment and detection have led to an increase in the number of long-term survivors [1,2]. Hence recent randomized cancer trials include QOL as an outcome measure [3,4], and intervention methods to enhance QOL have been articulated [5,6].

The issues of concern include, the impact of cancer on the QOL of the patient and family caregiver [7-12], the relationship with socio-demographic variables [13-15], side effects of treatment [16-19], and how patient-caregiver characteristics interact to affect the QOL of the patient-caregiver dyad [20-25]. These issues are related to the nature of the disease. For example, recent studies indicate that clinically severe anxiety, depression and fatigue, prevalent in at least one-fifth of cancer patients, do predict poor QOL [26-28]. In the case of family caregivers, the burden of caring for their relatives [29] is associated with significant levels of anxiety and depression [30,31]. The problematic issues for patients include sexual dysfunction associated with treatment, body image, fears over child bearing potential, and maintaining a household and career [32-35]. Hence, in the two or three years following diagnosis and treatment, women with breast and gynecologic cancers have significantly lower QOL domain scores than matched general population groups [8,36,37]. However, the robust finding from longitudinal studies is that, majority of long-term (≥ 5 years) survivors have QOL domain scores that are either similar to or higher than the general population[7,9-11]. The findings for the association of socio-demographic characteristics (especially age and education) with QOL are conflicting [6,9,13,15,38].

For chemotherapy, QOL decreased up to six months after treatment[19,36,38], but tended to return to normal levels at 12 months [16,22,39,40]. It appears that radiotherapy has a negative and chronic impact on QOL [17], and that surgical patients fare better than those who receive radiotherapy [41].

These QOL issues in cancer care have rarely been tested in developing countries where a rising incidence of breast and gynecologic cancers has been noted [29,32,42,43]. Sudan, a country in north Africa (population: 35 million), is typical of a developing country that has recently articulated a National Cancer Control Program (NCCP) in collaboration with the WHO [44]. We considered that it would be an ideal place to test the QOL issues highlighted above, with a view to enhancing the quality of delivery of the NCCP. According to statistics from the country's Ministry of Health, cancer is now the third leading cause of death, after malaria and viral pneumonia, accounting for 5% of all deaths. The commonest cancers in women are those of the breast, cervix and ovary, with breast and cervical cancer accounting for 50% of all cancers among Sudanese women [44]. The main radiotherapy center is at the Radiation and Isotope Center, Khartoum (RICK).

This report is based on subjects assessed at RICK, Khartoum, in 2005/2006.

Our review of the psycho-oncology literature as it pertains to QOL showed that researchers seemed to have paid scant attention to the following areas: First, the few studies on family caregiver QOL [20-25,45] did not assess the impact of caregiver impression of patient's QOL on the QOL of the patient and that of the caregiver. Furthermore, there is paucity of information on the relationship of patient's variables with the caregiver's QOL. This perspective is important because the psychological literature on "expressed emotions" (i.e., the impact of emotional interactions in the family on clinical outcome) has consistently shown that family caregiver's emotional appraisal of the patient has an impact on clinical outcome [46]. Katschnig [47] has suggested that it is necessary to involve family members for additional views on aspects of QOL. Second, the relatively few studies that compared QOL across female cancer types [8,14,24,48-50] did not compare women with breast and gynecologic cancers. In addition, there is a paucity of studies that compared the QOL of women with cancer and those with other chronic medical illnesses. Our study was designed to help fill these gaps in knowledge.

The objectives of the study were as follows:

- to compare the subjective QOL of Sudanese women living in stable condition with breast, cervical and ovarian cancer and those of their family caregivers, using the WHO 26 – item Quality of Life Instrument (the WHOQOL – Bref);

- to compare their ratings with those of a socio-demographically matched general population sample, as well as diabetic and psychiatric women patients similarly assessed in previous studies in Sudan [51-54];

- to assess the association of patient's QOL with socio-demographic variables, age at onset of illness, duration of illness, as wells as treatment with chemotherapy and radiotherapy;

- to examine the association of patient's demographic and clinical characteristics with the QOL of the family caregiver

- to examine the concordance between the QOL ratings of the patients and the family caregivers' ratings (or impressions) of the patients' QOL;

- to assess the characteristics of the patient, illness and family caregiver that can predict the patient's and caregiver's subjective QOL.

Based on our previous findings and evidence from the literature, we hypothesized as follows: First, most patients would be satisfied with items related to family supports and general well being; but not with items related to the poor national economy. There would be no significant differences in QOL domain scores between the group of cancer patients and between the family caregiver groups. While cancer patients and their caregivers would have significantly lower QOL scores than the general population, they would have similar scores with diabetic and psychiatric patients in the same cultural setting. Second, socio-demographic variables would have no significant association with QOL. Furthermore, patients with shorter duration of illness and currently on radiotherapy would have lower QOL scores.

Third, patient-caregiver characteristics would mutually interact and be associated with each other's QOL. In addition, there would be highly significant concordance between patient's ratings and caregiver's ratings of the patient. Fourth, the most significant predictor of the patient's and caregiver's QOL would be the caregiver's impression of the patient's QOL [51-54].

The clinical relevance of these hypotheses is that they could help to define a subset of stable patients and their caregivers whom clinicians need to give focused attention, and identify the patient and family characteristics which can be tapped as adjuncts to drug treatment to make for better quality of care.

## Methods

### Operational definitions

We accepted the WHO definition of QOL as individuals' perception of life in the context of the culture and value system in which they live and in relation to their goals, expectations, standards and concerns[55]. Our focus was on subjective QOL, as distinct from objective QOL[56]. We defined subject's satisfaction as the level of positive appreciation for each item. We quantified each group's satisfaction with each item as at least 50% of respondents in the group positively appreciating the item (i.e., proportion of subjects in the group rating satisfaction for the item as "satisfied" or "very satisfied"); dissatisfaction (< 50%); bare satisfaction (50 – 65%); moderate satisfaction (66 – 74%); and highest satisfaction (≥ 75%)[51,56].

#### The setting

The RICK, Sudan, has specialist staff that provide chemotherapy, radiotherapy, hormonal nuclear diagnosis and treatment for about 3500 new patients yearly. From 1967 to 2003, there was a ten-fold increase in the number of attendees. The daily out-patient clinic, where the study took place, is run in rotation by 15 medical consultants and 12 registrars. The cost of treatment for cancer patients is highly subsidized by a charity fund (Zakat or alms) and an insurance scheme. By a special arrangement unique to RICK, these funding agencies attempt to provide free services for all cancer patients.

#### Subjects

The patients were consecutive outpatient clinic attendees who fulfilled the study's inclusion criteria. First, the patients had been formally diagnosed for at least one year, had received relevant treatment (surgery/chemotherapy/radiotherapy) and were now attending the clinic for routine follow-up or completion of radiotherapy or chemotherapy. That is, they were in stable clinical condition at the time of interview. Second, each patient was accompanied by at least one family member or friend who lived with her, was responsible for caring for her at home and could complete the questionnaires in Arabic. It should be noted that in the traditional extended family system where the patients lived, care giving roles are shared by several people in the household [57].

The general population groups were selected by quota sampling from our WHOQOL-Bref data base for Sudan[51], to match the patients and caregivers by gender, age, and level of education.

### The WHOQOL – Bref

This is a 26-item self-administered generic questionnaire, a short version of the WHOQOL – 100 scale[55]. It emphasizes subjective experiences (i.e., subjective QOL) rather than objective life conditions (or objective QOL)[56]. It was developed in a wide range of cultural and clinical settings, including oncology. It is made up of domains and facets (or sub – domains). Domains are broad groupings (e.g., physical/psychological) of related facets. The items on "overall rating of QOL" (OQOL) and subjective satisfaction with health, are not included in the domains, but are used to constitute the "general facet on health and OQOL". There are two models of the WHOQOL – Bref. One model has six domains, namely, physical health, psychological health, level of independence, social relationships, environment, and spiritual. To derive the second (4 – domain) model, the domain of level of independence was merged with that of physical health, while the "spiritual" domain was added to the psychological.

### Modification of the WHOQOL – Bref for the impression of caregivers

In order to produce the version of the WHOQOL – Bref with which the family caregivers rated their impression of the patients' QOL, we used the method of Sainfort et al[58], by giving a new direction to each item, so that the caregiver could rate the patient as an observer. The modification of the WHOQOL – Bref was thus minimal[51].

The internal consistency of the WHOQOL – Bref, as assessed by Cronbach's alpha coefficient for the responses of all subjects, was very high for the patients (0.92), the caregivers' impressions (0.88), the family caregivers (0.92) and the general population (0.91).

#### Data collection procedure

The questionnaires were translated into Arabic by the method of back – translation and have been used in recent studies in Sudan to assess psychiatric and diabetes patients and their family caregivers [51-54]. Written permission to carry out the study was obtained from the authorities of the hospital. The patients and their family caregivers gave consent to participate after the objectives of the study were explained to them. In a pilot exercise, the instrument was found to be suitable to the cultural setting[51].

All questionnaires were administered by a female social welfare staff of the center. In the preliminary stage of the study, the research assistant was trained in the use of the questionnaires using patients who did not participate in the main study. The study commenced when the research team was satisfied that the research assistant could confidently administer the questionnaires to patients. Patients and caregivers completed the questionnaires privately and without interference from the research assistant, after clarification of the objectives of the study and the meaning of the items. Illiterate patients were assisted by their educated relatives to complete the questionnaire, after the caregiver had completed his or her own. Literacy in Arabic language is very high in Sudan, as it is the language of the Holy Koran. The study was carried out in compliance with the Helsinki Declaration. Hence, ethical approval for the work was obtained from the following institutions: The Radiation and Isotopes Centre, Khartoum, Sudan; the University of Ribat – Criminology and Social Studies Research Institute, Khartoum, Sudan; and the Faculty of Medicine, Kuwait University. In addition, patients and their family caregivers gave verbal informed consent to participate in the study. They were duly informed that there would be no negative consequences for declining to participate. As is well known in our culture for such non-invasive studies[51], all families approached freely consented to participate in the study, especially as the approach was made by clinic staff in charge of the cases.

The physician in-charge of each case assisted the research assistant to record the relevant clinical data.

#### Data analysis

Data were analyzed by the SPSS – version 12. For the first hypothesis, the pattern of frequency counts was used to assess group satisfaction with QOL items. Summary scores were generated by organizing the items of the WHOQOL-Bref into the six domains and four domains previously highlighted. We compared mean differences in domain scores for the three cancer groups and for the three family caregiver groups by either one-way ANOVA or Kruskal-Walli's chi-square, depending on whether the data were normally or not normally distributed. Domain scores of the patients (as a group) were compared with those of the matched general population group and published mean scores for women with diabetes and psychiatric illnesses from Sudan [51-54] (using independent sample t-test or Mann-Whitney U test), after controlling for differences in socio-demographic characteristics. A similar analysis was done for the caregivers' data. Domain scores of the patients (as a group) and caregivers (as a group) were compared by paired t-test. For the second hypothesis, the relationship between age, duration of illness and QOL domain scores was assessed by Pearson's correlation and Spearman's correlation. The association between other socio-demographic variables (level of education, occupation and marital status) and QOL was assessed by one-way ANOVA. In view of the fact that a number of the socio-demographic variables were significantly associated with QOL domain scores in these uni-variable analyses, analysis of covariance (ANCOVA) was used to assess the socio-demographic variables associated with QOL domain scores in multivariate relationships. The association between chemotherapy/radiotherapy and QOL domain scores was assessed by t-tests and one-way ANOVA. For the third hypothesis, caregiver QOL domain scores were analyzed to see how these scores related to patient's socio-demographic and clinical (e.g., being on chemotherapy or not, and duration of illness) characteristics, as well as caregiver relationship to the patient, using t-tests, one-way ANOVA and Pearson's correlation as outlined above for the data of patients. Furthermore, the concordance between patient's WHOQOL-Bref ratings and the impression of the caregiver was assessed in two ways. First, we used Kendall's tau to examine the correlation between the corresponding domain scores (Kendall's tau was preferred because it is more conservative than Pearson's correlation, as it takes ties into consideration). Second, we used intra-class correlation to examine the internal consistency of the patient-caregiver rating. For the fourth hypothesis, the predictors of patients' QOL and caregivers' QOL (based on patients' general facet and caregiver's general facet, as dependent variables) were assessed in step-wise regression analysis. Missing data were handled by excluding cases analysis by analysis. All tests were two-tailed. A Bonferroni correction (P = 0.01) was used for multiple tests; otherwise, the level of statistical significance was set at P < 0.05. The results of the parametric analysis (t-test, ANOVA, etc), being more conservative, are presented because they were similar to the non-parametric equivalent results.

## Results

### Socio-demographic characteristics (Tables 1A &1B)

**Table 1A:** Socio-demographic characteristics of patients by diagnosis of patient Vs general population control group

Characteristics	Ca Breast	Ca Cervix/Uterus	Ca Ovary	X^2 ^(F)	Df	P	All cancer groups	General population	P
	N = 117	N = 46	N = 18				N = 181	N = 177	

Pt gender: M/F (%)	0/117	0/46	0/18				0/181	0/177	
Age of pt (SD)	43.0(10.0)	51.1(12.3)	37.7(11.8)	13.2	2/178	0.0001	44.6(11.5)	44.6(11.7)	ns
Single/married (%)	24(20.5)/74(63.2)	2(4.3)/25(54.3)	4(22.2)/14(77.8)	20.8	4	0.0001	30(16.6)/113(62.4)	-	
Unemployed/student(%)	95(82.6)/7(6.1)	41(93.2)/1(2.3)	14(82.4)/2(11.8)				150(82.9)/9(4.9)	-	
Medium & high skill	13(11.3)	2(4.5)	1(5.9)	4.3	4	ns	16(8.8)	-	
Illiterate/primary (%)	22(19.5)/54(47.8)	23(50.0)/12(26.1)	4(22.2)/5(27.8)	18.6	4	0.001	120(67.8 of 177)	107(60.5)	0.18
High schl & ollege	37(32.7)	11(23.9)	9(50.0)				57(32.0 of 177)	70(57.5)	
Age at onset(SD)	39.9(9.9)	48.0(12.0)	34.1(0.6)	14.5	2/178	0.0001	41.4(11.4)		
Duration illness (yrs)	3.2(2.3)	3.1(2.4)	3.6(5.2)			ns	3.2(2.7)		

**Table 1B:** Socio-demographic characteristics of family caregivers by diagnosis of patient Vs general population control group

Characteristics	Ca Breast	Ca Cervix/Uter	Ca Ovary	X^2 ^or (F)	Df	P	All cancer groups	General population	P
	N = 117	N = 46	N = 18				N = 181	N = 181	

Carer: M/F (%)	76(65.0)/41(35.0)	23(50.0)/23(50.0)	14(77.8)/4(22.2)	5.2	2	0.08	113(62.4)/68(37.6)	112(62.4)/69(38.1)	
Carer's age(SD)	43.1(12.7)	41.8(15.4)	46.4(9.4)	0.8	2/178	ns	43.1(13.1)	43.0(13.1)	ns
Carer illiterate/primary schl (%)	18(15.4)/25(21.4)	11(23.9)/13(28.3)	3(16.7)/5(27.8)	3.5	4	ns	75(41.4)	63(34.8)	
High schl & ollege	74(63.2)	22(47.8)	10(55.6)				106(58.6)	118(65.2)	ns
Single/married(%)	35(29.9)/76(65.0)	21(45.7)/23(50.0)	2(11.1)/16(88.9)	9.4	4	0.05			
Unemployed/student(%)	33(33.7)/31(31.6)	19(42.2)/16(35.6)	5(27.8)/4(22.2)	4.9	4	ns			
Medium & High skill	34(34.7)	10(22.2)	9(50.0)						
Carer: parent/sibling(%)	10(8.5)/35(29.9)	2(4.3)/17(37.0)	1(5.6)/6(33.3)	16.5	8	0.04			
Carer: spouse (%)	38(32.5)	7(15.2)	10(55.6)						

The 181 patients consisted of 117(64.6%) women with breast cancer, 42(23.2%) with cervical cancer, 4(2.2%) with endometrial cancer and 18(9.9%) with ovarian cancer, mean age 44.6 years. (Note: For the purpose of analysis, data for the cervical and endometrial cancer cases were merged, because of the small number of the later. Hence we have 46 cases of cancer of cervix and uterus. Henceforth, we refer to this group as "cervical cancer"). Cervical cancer patients were significantly older than others (P < 0.0001). The patients were predominantly married (62.4%), formally not employed (i.e., housewives, 82.9%), and only 31.5% had up to high school education. They had been ill for 3.2 (SD2.7) years. Although the cervical cancer patients were significantly more likely to be divorced or widowed (P < 0.001), there were no significant differences in occupation and education between the groups of patients (P > 0.05). While those with cervical cancer were significantly older at onset of illness than the others (P < 0.0001), there were no significant differences in duration of illness (P > 0.05).

Tables 1A and 1B show that the patients and caregivers were well matched with their respective general population control groups by gender, age and education (P > 0.05).

Their family caregivers consisted of 113 (62.4%) men and 68 (37.6%) women, mean age 43.1 years (Table 1B). The caregivers were predominantly married (63.5%), either unemployed or in low skill occupation (55.3%), and 106 (58.6%) attained at least high school education. There were no significant differences in socio-demographic variables for the caregiver groups. There were 55 (30.4%) spouses.

### Satisfaction with items of QOL: (Tables 2A &2B)

**Table 2A:** Comparative level of group satisfaction with QOL items: for patients and caregivers' impression of patients' QOL

Highest satisfaction: ≥ 75% subjects	Moderate satisfaction: 66–74% subjects	Bare satisfaction: 50–65% subjects	Dissatisfied: < 50% subjects
	1. Patients with breast, cervical	and ovarian cancer	
Overall QOL(79%), feeling pain (87%), med treatment(75%), life meaningful (82%), ability concentrate(96%), physical environ(84%), energy(95%), bodily appearance(92%), ability to get around(98%), sleep satisfaction(91%), ADL(81%), work capacity(80%), self satisfaction(82%), personal relations (77%), friends' support(79%), living place(81%), negative feelings(85%)	Health satisfaction (69%), enjoy life (72%), access to health service (72%)	Feeling safe(64%), satisfaction with sex (53%)	Money for needs(39%), information available(35%), leisure opportunity (28%), transport satisfaction (33%)
	2. Caregivers' impression of breast/	cervical ovarian cancer	patients' QOL
Overall QOL(89%), health satisfaction (85%), life meaningful(96%), concentration(96%), physical environ(84%), energy(93%), bodily appearance(94%), getting around(97%), sleep (80%), ADL (79%), work capacity(80%), self satisfaction (90%), personal relations(80%), friends' support(82%), living place(85%)	Feeling safe(67%), access to health service (71%)	Feeling pain(64%), satisfaction with sex(55%)	Dependence on med treatment (25%), enjoy life(5%), money (41%), information availability (33%), leisure opportunity (28%), transport (28%), negative feeling (9%)

**Table 2B:** Comparative level of group satisfaction with QOL items: Family caregivers

Highest satisfaction ≥ 75% of subjects	Moderate satisfaction: 66 – 74% of subjects	Bare satisfaction: 50 – 65% of subjects	Dissatisfied < 50% of subjects
	3. Family caregivers	of patients with breast,	cervical and ovarian cancer
Overall QOL(99%), health satisfaction (93%), feeling pain(94%), med treatment (92%), enjoy life(94%), life meaningful (96%), concentration ability(99%), feeling safe(83%), physical environ(93%), energy (98%), bodily appearance(98%), getting around(99%), sleep satisfaction(99%), ADL (93%), work capacity(93%), self satisfaction (93%), personal relations(92%), friends' support(98%), living place satisfaction (90%), access to health service (77%)	Satisfaction with sex (66%)	Availability of health information (53%)	Money for needs (49%), leisure opportunity (49%), transport satisfaction (41%)

Using the operational definition for group satisfaction with QOL items, Tables 2A and 2B show that the patients and caregivers were generally highly satisfied with majority of the items. The interesting pattern that emerged was that the few areas of dissatisfaction concerned items related to their poor national material circumstance, viz: money for needs, availability of information about their health problems, opportunity for leisure, and transportation. On the other hand, the subjects expressed high levels of satisfaction with items related to their personal strengths (e.g., overall QOL, life meaningful, self satisfaction, lack of negative feelings), and available social support (e.g., support from friends, personal relations, place of living). The caregivers' ratings of patients were remarkably in agreement with patients' ratings.

### Differences in QOL domain scores between the groups: association with caregiver relationship to patient, and comparison with control group and caregiver impression: Table 3

**Table 3:** Differences in QOL domain scores for patient, family caregiver groups and general population control groups

	Mean (SD)							
Domains of WHOQOL-Bref	Ca breast N = 117	Ca Cervix N = 46	Ca ovary N = 18	P	All Ca groups N = 181	General Popn group N = 177	T	P

A: For patients								
Physical health 6-domain	13.6 (1.4)	12.9 (2.3)	13.4 (1.1)	ns	13.4 (1.6)	10.8 (2.6)	11.3	0.001
Psychological health 6-domain	21.3 (2.5)	20.1 (3.8)	20.9 (2.9)	ns	20.9 (2.9)	17.9 (3.6)	8.6	0.001
Independence	17.6 (2.2)	16.5 (3.1)	16.8 (2.2)	ns	17.2 (2.5)	14.7 (3.6)	8.2	0.001
Social relations	12.3 (2.9)	11.9 (3.2)	11.6 (3.6)	ns	12.1 (3.0)	11.0 (2.4)	3.4	0.001
Environment	30.6 (5.1)	29.5 (6.4)	29.9 (5.1)	ns	30.3 (5.4)	24.9 (5.5)	9.1	0.001
Spiritual	4.5 (0.8)	4.3 (1.1)	4.2 (1.1)	ns	4.4 (0.9)	3.9 (1.1)	4.9	0.001
General facet on health & QOL	8.4 (1.8)	7.8 (2.4)	8.4 (1.7)	ns	8.3 (1.9)	7.2 (1.8)	5.3	0.001
Physical health 4-domain	31.2 (3.4)	29.4 (5.3)	30.3 (3.1)	ns	30.7 (4.0)	25.6 (5.7)	9.9	0.001
Psychological health 4-domain	25.9 (3.1)	24.5 (4.7)	25.1 (3.8)	ns	25.4 (3.7)	21.8 (4.3)	8.2	0.001
								
B. For caregivers of the patients								
Physical health 6-domain	14.3 (1.2)	14.4 (1.2)	14.7 (0.8)	ns	14.4 (1.1)	11.3 (2.4)	15.7	0.001
Psychological health 6-domain	22.1 (1.7)	21.8 (1.9)	22.2 (1.5)	ns	22.0 (1.8)	18.8 (3.5)	10.8	0.001
Independence	18.9 (1.9)	18.8 (2.2)	19.1 (1.6)	ns	18.9 (1.9)	15.3 (3.0)	13.5	0.001
Social relations	13.4 (2.0)	13.4 91.9)	13.6 (2.4)	ns	13.4 (2.0)	11.6(2.4)	7.6	0.001
Environment	32.9 (4.6)	32.3 (5.3)	30.8 (3.4)	ns	32.5 (4.7)	25.9 (5.9)	11.5	0.001
Spiritual	4.7 (0.5)	4.7 (0.6)	4.5 (0.6)	ns	4.7 (0.5)	4.0 (0.9)	8.7	0.001
General facet on health & QOL	9.3 (1.1)	9.1 (1.2)	9.4 (1.1)	ns	9.3 (1.1)	7.6 (1.7)	11.3	0.001
Physical health 4-domain	33.2 (3.1)	33.2 (3.3)	33.8 (2.2)	ns	33.3 (3.0)	26.6 (4.9)	15.5	0.001
Physical health 4-domain	26.8 (2.1)	26.5 (2.5)	26.7 (1.9)	ns	26.7 (2.2)	22.8 (4.1)	11.1	0.001

Note: In all the domains, caregivers had significantly higher scores than the patients (t ranged from 3.9 to 7.3, df = 360, P < 0.0001).

In all the domains, the three groups of cancer patients had similar scores (P > 0.05). Similarly, there were no significant differences for the corresponding family caregiver groups (P > 0.05). The caregiver ratings of the patients were in the same direction, except for the independence domain, where patients with cancer of the ovary were rated as having significantly higher scores than those with cervical cancer (F = 4.9, df = 2/177, P = 0.008). In addition, spouses (all men) rated the patients as having a higher QOL than the parents rated them in the social relations domain (P < 0.0001).

Similarly, there was a tendency for patients being cared for by their spouses to have the highest scores. This trend reached significance for only the social relations domain (F = 7.9, df = 4/176, P < 0.0001).

For the caregivers, there was a tendency for parents to have the lowest QOL scores. This reached significance for physical health (P < 0.0001); psychological health (P < 0.0001); independence (P < 0.0001); and general facet (P < 0.0001).

In view of the similarity of QOL domain scores among the cancer groups and among the caregiver groups, we used the total group scores of the patients and caregivers to compare with their respective matched general population groups.

In all the QOL domains, the patients and family caregivers had much significantly higher scores than corresponding matched general population control groups (P < 0.001) (Table 3). In addition, the patients had significantly higher scores than psychiatric(N = 136) and diabetic (N = 111) women patients in Sudan who were similarly assessed, even after controlling for socio-demographic differences (t ranged from 5.7 to 14.1, P < 0.0001). In all the domains, caregivers had significantly higher scores than the patients (P < 0.0001).

### Association of socio-demographic variables with QOL domain scores (Table 4)

**Table 4:** Association of patient and caregiver socio-demographics with QOL: significant covariates of QOL in ANCOVA

Socio-demographics as covariates	Pts' QOL domains affected	F	P
A: For patients			
Marital status: married > single	Social relations Spiritual	16.7 6.8	0.001 0.01
Occupation: medium/high skill > unemployed	Psychological health 6-domain Spiritual General facet on health & QOL	3.8 3.8 5.5	0.05 0.05 0.02
Education: high school/college > illiterate	Environment	4.9	0.03
Education of caregiver	Environment	4.9	0.03
B. For caregivers of patients			
Marital status of patient	Physical health 6-domain	5.2	0.02
Education of patient	Spiritual	7.3	0.008
Age of patient	Psychological health 6-domain Spiritual	6.5 6.6	0.01 0.01
Education of caregiver	Physical health 6-domain Psychological health 6-domain Independence Environment Spiritual	7.7 10.5 7.6 29.4 10.9	0.006 0.001 0.007 0.001 0.001
Marital status of caregiver	Physical health 6-domain Independence Social relations		
Occupation of caregiver	Physical health 6-domain Psychological health 6-domain		
Age of caregiver	Physical health, physical health, independence, social relations, general facet	6.3 – 30.9	0.001
Caregiver's sex	Psychological health, social relations, environment, general facet	5.6 – 15.2	0.02 – 0.001

In multivariate analysis, where all the socio-demographic variables (patients' and caregivers') were simultaneously entered in ANCOVA as covariates, and QOL domain scores as dependent variables, we found that the significant covariates for patients were marital status, occupation and education of the patient, as well as education of the caregiver. The pattern that emerged was that, higher QOL of scores for patients were associated with patient being married, employed in medium skill/high skill occupation, and having attained at least high school education.

When the multivariate analysis was done using the caregiver's QOL domain scores as dependent variables, the following patterns emerged. First, the patient's socio-demographic variables were significantly associated with the caregiver's QOL domains. Hence, the following patient characteristics were associated with higher caregiver QOL: patient being married, older, and with at least high school level of education. Second, caregivers who were male and older had much significantly higher QOL scores in most of the domains (P mostly < 0.01). Third, the following caregiver characteristics were associated with higher caregiver QOL scores: at least high school education, being married and engaged in medium skill/high skill work (P mostly < 0.01). In other words, the patient's socio-demographic variables were more frequently associated with the caregiver's QOL than the caregiver's socio-demographics were associated with the patient's QOL (see Table 4).

### Association of clinical variables with QOL domain scores (Tables 5, 6, 7)

**Table 5:** Correlation of duration of illness with QOL domain scores (Pearson's r)

Correlations with pt's QOL ratings	Pearson's r	P	Correlations with caregiver impression of pt's QOL	Pearson's r	P
Duration of illness Vs			Duration of illness Vs		

Physical health (N = 180)	0.29	0.000	Caregiver impression pt's physical health (N = 180)	0.22	0.003
Psychological health (N = 180)	0.29	0.000	Caregiver impression pt's psychol health (N = 179)	0.19	0.008
Independence (N = 181)	0.23	0.001	Caregiver impression pt's independence (N = 180)	0.17	0.02
Social relations (N = 179)	0.15	0.04	Caregiver impression pt's social relations (N = 179)	0.15	0.04
Environment (N = 180)	0.26	0.000	Caregiver's impression pt's environment (N = 178)	0.22	0.003
General facet on health & QOL (N = 180)	0.16	0.03	Caregiver's impression pt's general facet (N = 180)	0.16	0.03

**Table 6:** Comparison of QOL domain scores: subjects feeling currently ill versus not feeling currently ill

	Mean (SD)		Statistics			Mean (SD)		Statistics		
QOL domains	Pt feels currently ill	Pt not feeling ill currently	T	Df	P	Carer feeling ill	Carer not ill	T	Df	P

	N = 97	N = 83				N = 98	N = 83			

Physical health	12.7 (1.7)	14.2 (1.1)	6.6	178	0.001	14.3 (1.2)	14.5 (1.1)			ns
Psychological health	19.8 (3.3)	22.4 (1.5)	6.7	178	0.001	21.9 (1.8)	22.1 (1.7)			ns
Independence	16.3 (2.7)	18.4 (1.8)	6.1	179	0.001	18.8 (2.0)	18.9 (1.8)			ns
Social relations	10.8 (3.2)	13.6 (1.9)	6.9	177	0.001	13.1 (2.2)	13.8 (1.8)	2.1	176	0.04
Environment	28.2 (5.3)	32.7 (4.6)	6.1	178	0.001	31.8 (4.7)	33.5 (4.4)	2.5	176	0.02
Spiritual	4.1 (1.1)	4.8 (0.5)	5.6	178	0.001	4.6 (0.6)	4.8 (0.5)			ns
General facet health & QOL	7.3 (2.0)	9.4 (1.2)	8.2	178	0.001	9.1 (1.2)	9.4 (1.0)			ns

**Table 7:** Relationship of chemotherapy and radiotherapy with QOL of patients

	Mean (SD)			Mean (SD)				
QOL domains	Subject on chemotherapy	Not on chemotherapy	P	Subject on radiotherapy	Not on radiotherapy	T	Df	P

	N = 140	N = 40		N = 137	N = 41			

Physical health	13.4 (1.6)	13.3 (1.7)	ns	13.5 (1.4)	12.9 (2.2)	2.3	176	0.03
Psychological health	21.0 (3.0)	20.8 (2.8)	ns	21.3 (2.4)	19.7 (4.1)	2.3	176	0.002
Independence	17.3 (2.5)	17.1 (2.6)	ns	17.4 (2.5)	16.7 (2.9)	1.6		ns
Social relations	11.9 (3.0)	12.8 (2.9)	ns	12.4 (2.8)	10.9 (3.4)	2.9	175	0.005
Environment	30.1 (5.5)	30.9 (5.5)	ns	30.7 (5.2)	28.9 (6.1)	1.9	176	0.06
Spiritual	4.4 (0.9)	4.5 (0.9)	ns	4.5 (0.8)	4.1 (1.1)	2.6	176	0.01
General facet on Health & QOL	8.3 (1.9)	8.2 (2.0)	ns	8.4 (1.9)	7.8 (2.3)	1.7		ns

#### Duration of illness (Table 5)

The duration of illness was significantly correlated with all the patient's QOL domain scores (P mostly < 0.01), except for the spiritual domain(P > 0.05). But patient's duration of illness was not significantly correlated with caregiver's QOL domain scores (P > 0.05). In line with the rating of patients, QOL domain scores derived from caregiver's rating of the patient were significantly correlated with patient's duration of illness in all domains (P mostly < 0.01), except for the spiritual domain (P > 0.05).

#### Subject currently feeling ill (Table 6)

In all the domains, the patients who felt currently well had much significantly higher scores than those who felt currently ill (P < 0.001). This trend was evident for the family caregivers, in which case the significant differences were noted for only the social relations (P = 0.04) and environment domains (P = 0.02).

### Association of chemotherapy and radiotherapy with QOL (Table 7)

While there were no significant differences in QOL domain scores between those currently on chemotherapy and those not on chemotherapy (P > 0.05), the patients on radiotherapy tended to have higher scores than those not on radiotherapy. This reached significance for the following domains: physical health, psychological health, social relations and spiritual (P mostly < 0.01). However, according to the caregivers' ratings of the patients, subjects on chemotherapy were judged to have higher scores (compared with those not on chemotherapy) for the psychological health domain (P = 0.009). In addition the caregivers rated patients on radiotherapy as having higher scores for the social relations domain (P = 0.006). There was a non-significant tendency for relatives of patients on radiotherapy to have higher QOL domain scores.

### Concordance of patients' ratings and caregiver impression patients' QOL (Table 8)

**Table 8:** Concordance of patients' rating of QOL and caregiver impression of patients' QOL

	Mean (SD)		Statistics				
QOL domains	Patient's rating QOL	Carer rating pt's QOL	Paired T	DF	P	Kendall's tau	P

Physical health	13.4 (1.7)	12.9 (1.7)	3.9	178	0.001	0.56	0.001
Psychological health	20.9 (2.9)	19.2 (1.6)	10.1	177	0.001	0.45	0.001
							
Independence	17.2 (2.5)	14.8 (1.5)	14.1	179	0.001	0.35	0.001
Social relations	12.1 (3.0)	12.2 (2.6)	0.5		ns	0.64	0.001
Environment	30.4 (5.4)	34.6 (5.6)	15.4	176	0.001	0.60	0.001
Spiritual	4.7 (0.5)	4.7 (0.5)	0.4		ns	0.12	ns
General facet on health & QOL	8.3 (1.9)	8.5 (1.4)	2.3	178	0.02	0.57	0.001

Intra-class correlation coefficient (ICC) for responses to the two questionnaires = 0.96 (95% C.I. = 0.95 – 0.97)

The patients rated themselves as having higher scores than the caregivers rated them, for the following domains: physical health, psychological health and independence (P < 0.001). However, the patients' own ratings and caregiver impression scores were highly significantly correlated (Kendall's tau mostly over 0.45, P < 0.001), except for the spiritual domain (P > 0.05). Furthermore, there was highly significant internal consistency between the ratings of the patients and the impression of the caregivers (intra – class correlation = 0.96; 95% C.I. = 0.95 – 0.97).

### Predictors of patients' and caregivers' QOL (Table 9)

**Table 9:** Predictors of QOL of patients and caregivers: dependent variables: general facet of patients and carers in multiple regression analysis

Dependent variable	Predictors or independent variables	Variance (%)	Total variance	Beta	T	P
General facet on health & QOL for pts with breast, cervical & ovarian cancer	General facet caregiver impression of pt	42.7	53.6	0.54	8.0	0.000
	Pt currently feels ill	9.1		0.30	4.9	0.000
	Duration of illness	1.8		0.14	2.3	0.02
General facet on health & QOL for caregivers of pts with breast, cervical & ovarian cancer	Caregiver currently feels ill	29.2	56.1	0.41	6.8	0.000
	General facet carer impression of pt	11.9		0.24	4.1	0.000
	Caregiver relationship to pt	5.6		0.16	2.6	0.01
	Caregiver's age	3.5		-0.27	-4.3	0.000
	Pt's age	2.7		-0.15	-2.6	0.01
	Caregiver gender	1.9		-0.17	-2.7	0.007
	Pt on radiotherapy	1.3		-0.12	-2.1	0.04

In multiple (step-wise) regression analysis with the general facet on health and QOL as the dependent variable, the most important predictor of the patient's QOL was the general facet derived from the family caregiver impression rating of the patient's QOL. This variable accounted for 42.7% of the variance. The other significant predictors were patient feeling currently ill (variance 9.1%) and duration of illness (variance 1.8%). In a similar analysis for the family caregiver, the caregiver impression general facet was an important predictor of the caregiver's QOL, accounting for 11.9% of the variance. The other significant predictors included caregiver feeling currently ill (variance 29.2%), and caregiver relationship to patient (variance 5.6%).

## Discussion

### Limitations and strengths of the study

The limitations of the study are that it was cross-sectional, from a single center, with a relatively small sample size for the ovarian cancer group, the patients were selected because they had family support, and we did not record tumor stage and degree of recovery from cancer. Although the proportion of breast, cervical and ovarian cancer cases in our sample is reflective of the prevalence of these conditions in Sudan [44], our findings cannot be generalized for this population of patients in the country because of the noted limitations.

However, we were able to compare three women cancer groups with their family caregivers and a socio-demographically matched general population group. In addition, we assessed the relationship of caregiver impression of the patient with the QOL of the patient and the caregiver. This is a rare methodology in the psycho-oncology QOL literature.

### Socio-demographic characteristics

On average, our patients and their caregivers (mean age 44.6, 43.1 yrs, respectively) were relatively younger than similarly assessed subjects in studies from the developed countries (generally over 54 yrs) [45,59]. But our patients were similar in age to breast and gynecologic patients from Africa[43,60,61]. This is in line with well known international age trends whereby the average age of subjects in developing countries is less than that in the developed countries. Also similar to reports from Africa, the patients were predominantly married, with lower levels of education, and not in formal employment. However, their family caregivers had better educational and employment attainments, perhaps because they were on average younger in age than the patients.

### Comparison of QOL item ratings and domain scores

In analyzing for the first hypothesis, we found that over two-thirds of the patients were highly satisfied (i.e., responded satisfied/very satisfied) with 17(65.3%) of the items of the WHOQOL-Bref. This is a high level of satisfaction with circumstances of living. It is noteworthy that the few items that they did not feel satisfied with were those that reflected the material poverty of their country, such as money for needs, availability of health care information, opportunity for leisure and transportation. The ratings of the family caregivers and the caregivers' impression of the patients were similar to those of the patients. This response pattern is an indication of the reliability of the ratings of the patients. It shows that in rating their subjective QOL, the patients and caregivers made realistic appraisals of their life circumstances.

In line with this high level of satisfaction, the patients had high scores for all the domains of QOL. With regard to the finding that our patients and their family caregivers had significantly higher QOL domains scores than matched general population groups, as well as diabetic and psychiatric patient groups in Sudan, we note that this is a fairly common finding in the literature for long-term survivors of cancer [7,9-11]. What is rather surprising in our report is that it is based on subjects from a developing country. We can only attempt to explain the relatively high subjective QOL scores for our subjects. First, our patients had been receiving formal treatment for averagely over three years. They were living in the community, and not in need of hospitalization or further surgery. Although we did not assess it specifically, clinical experience shows that the patients were not abusing alcohol (forbidden by the culture) or other drugs of dependence. Second, the patients were chosen because they had evident social support from their families. Third, the RICK has an active system of financial support for needy patients, such that over a third of such patients received full re-imbursement while the rest received significant support [44]. This level of physical well-being, effective social support and lack of drug abuse has been associated with good subjective QOL among cancer survivors [1,2,7,62]. In a report from South Africa, it was noted that lower QOL among cervical cancer women was associated with inpatient status, more advanced stage of the disease, and lower physical performance scores, among other factors[62]. Since our patients were negative for these factors, we speculate that the burden of care would have been relatively light for the family caregivers, and hence their QOL would not be significantly diminished.

In accounting for the high subjective QOL among cancer patients and their family caregivers, the recurring themes in the literature are the role of religion/spirituality, resilience, " fighting spirit", "helplessness/hopelessness", active coping and "hardiness"[52,59,62-66]. The popular impression is that surviving the cancer experience changes the views of patients and families on life and relationships in an overwhelmingly positive way [66], and they predominantly resort to spirituality/religion as a way of coping[63]. Although we did not assess these issues, it is easy to see how the religious culture in which our subjects lived could have contributed to these inner strength-enhancing values among them.

The clinical implication of this finding of high subjective QOL among our patients and their caregivers is that it could be used in health education activities to reduce the popular impression of cancer as an inevitable killer disease, to encourage patients to report early for treatment, to patronize cancer screening programs, and to show that effective treatment of cancer can lead to a restoration of QOL.

### Socio-demographic and clinical factors associated with QOL; patient-caregiver concordance of ratings

Our second and third hypotheses were partially upheld. First, although age was not significantly associated with patients' QOL; higher QOL was associated with patients being married, as well as having higher levels of employment and higher educational attainments. While there are no consistent findings on the role of these socio-demographic factors [6,9,13,15,38] it is reasonable to assume that marriage, higher educational attainment and employment will increase the potential for awareness of disease, social support and the use of positive coping methods, all of which can contribute to higher QOL. Accordingly, education was the only caregiver characteristic that had a significant association with patient's QOL.

Second, Table 4 shows that patients' socio-demographic characteristics were significantly associated with some QOL domains of family caregivers. Hence, caregivers of patients who were married, older and better educated tended to have higher QOL scores. Similarly, caregivers who were men, older, married, educated, and formally employed had significantly higher QOL. With regard to caregiver relationship to the patient, we found that patients who were being cared for by their spouses tended to have higher scores than those who were being cared for by their parents, and that parents tended to have the lowest scores.

These findings show the importance of considering the interaction of patient-caregiver characteristics in cancer care[20,45]. The indication is that families living with cancer are vulnerable if the patient is less educated, single, not formally employed, and the caregiver is female, parent, younger, less educated and unemployed [67]. Such patient-caregiver dyads need to be singled out for relevant social support by the clinical team, in order to enhance the quality of care.

Third, Tables 5 and 6 show that higher QOL for the patients and caregivers was highly significantly associated with patients' duration of illness and whether the subject was feeling currently well. These add to the list of vulnerability factors for the clinical team. The literature shows that cancer patients and their relatives experience their lowest QOL when the disease is newly diagnosed [8,36,37]. It appears that for those who are successfully treated, patients and caregivers become experienced in managing problems, especially where there is social support, leading to improvement in QOL[10-12,21].

Fourth (Table 7), while the non-significant relationship of chemotherapy with QOL has support in the literature[16,22,39,40] our finding of significantly higher scores for those currently on radiotherapy is at variance with the literature[17,41]. We suggest that a possible reason for this finding is the feeling of gratitude that the patients had for having the opportunity to receive this rare treatment in a country where there are only two such facilities for a population of over 30 million. This explanation is supported by the finding that caregivers of patients on radiotherapy also tended to have higher QOL than caregivers of patients not on radiotherapy.

Fifth, the high degree of concordance for the patient-caregiver dyad ratings (Table 8) at the mega-variable or macro level of domains (P < 0.001), and from the perspective of internal consistency (intra-class correlation = 0.95) is in support of our hypothesis, and is in line with the few available literature on the matter[22,24,53]. On the other hand, the concordance of patient-clinician rating tends to be low and not significant [68]. At one level, this supports the reliability of the responses of the patients. At another level, it shows that these caregivers did share the sufferings and hopes of the patients, and that they exhibited a sensitive empathy or what has been called "social intelligence" [69] with regard to the patients' condition. These factors promote high QOL for the patient and the caregiver[69].

### Predictors of patients' and caregivers' QOL

The finding that caregiver impression was a highly important predictor of the QOL of the patient and the caregiver, is in support of our fourth hypothesis. This finding has also been replicated in studies of psychiatric and diabetic populations in Sudan, and therefore merits attention [51-54]. In the case of patients, it is possible to explain this finding from the perspective of high concordance of the patient-caregiver ratings. However, such an explanation would not suffice for the successful prediction of the caregiver's QOL. For one thing, the caregivers had significantly higher scores than the patients in all the domains (P < 0.0001). Hence, even if they shared the same world view, that would not explain why the caregiver's rating of the patient would be a significant predictor of the caregiver's QOL. However, "expressed emotions" research in psychiatry has consistently shown that negative attitudes of family members do adversely affect clinical outcome[46], and it has been shown that patient-caregiver social and clinical characteristics do interact to affect each other's QOL in cancer[20,24,45,53]. We suggest that in the same way that family caregiver adverse emotional reactions have been found to predict relapse for severe psychiatric illnesses [46], caregiver positive appreciation of the patient's QOL could impact on the QOL of the patient and that of the caregiver. We suggest that recent brain-behavior findings about "mirror neurons"[70] and the phenomenon of "social intelligence" indicate that the patient-caregiver dyad interaction and its association with QOL has roots in the neurology of human behavior[69,70]. In reviewing these findings, Goleman noted that, "we are wired to connect", resulting in a brain-to-brain linkage in a mutually reverberating state which neuroscientists call " empathic resonance". " Our nervous systems are constructed to be captured by the nervous systems of others, so that we can experience others as if from within their skin. At such moments we resonate with their experience and they with ours" (pg 43)

The other significant predictors of QOL, especially the subject feeling sick, and caregiver relationship to the patient, are indicative of additional indices of vulnerability. The impact of psychic distress and fatigue on QOL in cancer is well documented [26-28]. In the case of caregivers, the implication of these vulnerability factors is that caregiving ability should not be taken for granted. If the call to involve families in the routine clinical management of patients succeeds, clinicians need to inquire into the ability of available family members to participate in the caregiving role at home. Our analysis indicates that caregivers who are parents and feel sick need specific attention from the clinical team [71].

## Conclusion

Our findings have added to the body of evidence that cancer patients in stable condition and with evident psychosocial support can hope to enjoy good QOL in the long term, if they remain in treatment. This is an evidence base that the nascent National Cancer Control Program (NCCP) in Sudan and other developing countries need to boost national health education about the impact of screening programs, modern treatments and social support on prognosis in breast and gynecologic cancers. Furthermore, the vulnerable groups that our analysis delineated indicate the characteristics of patients and family caregivers that the clinical team needs to pay particular attention to, in order to enhance the quality of care. The indication is that families living with breast and gynecologic cancer patients are vulnerable if the patient is recently diagnosed, less educated, single, not formally employed; and the caregiver is female, parent, younger, less educated, unemployed and feels sick. In this regard, the demonstrated interaction of patient-caregiver characteristics in this and other studies indicates that in the care of chronically ill patients, the patient and caregiver need to be considered as a unit for attention in the clinical setting. In particular, the finding of the predictive power of the caregivers' impression on the QOL of the patient and caregiver, shows that clinicians need to invest in the education and support of family caregivers in order to enhance their caregiving role in the "invisible health care system"[72].

## Competing interests

The author(s) declare that they have no competing interests.

## Authors' contributions

AWA and JUO jointly designed the study, analyzed the data and wrote up the manuscript. AWA trained and supervised the research assistant in Sudan. AG helped in the analysis and write-up of the manuscript. AOK and HMH supervised the interviews, ensured correct diagnosis and other clinical data, and critically reviewed the manuscript for intellectual content. AJ played an invaluable role in data analysis and interpretation of data. All authors read and approved the manuscript

## Pre-publication history

The pre-publication history for this paper can be accessed here:


